# Ambipolar field role in formation of electron distribution function in gas discharge plasma

**DOI:** 10.1038/s41598-017-15073-6

**Published:** 2017-11-03

**Authors:** Chengxun Yuan, E. A. Bogdanov, A. A. Kudryavtsev, K. M. Rabadanov, Zhongxiang Zhou

**Affiliations:** 1 Department of Physics, Harbin Institute of Technology, Harbin, 150001 China; 2 Saint Petersburg State University, St. Petersburg, 199034 Russia

## Abstract

It is shown that the local approximation for electron distribution function (EDF) determination at plasma periphery, where the ambipolar field is dominant, is not applicable even at high pressures when the characteristic plasma size exceeds the energy relaxation length of the electrons *R* > *λ*_*ε*_. Therefore, consistent results can be obtained only when solving the complete kinetic equation in both energy and spatial variables (i.e. it is necessary to solve nonlocal kinetic equation).

## Introduction

Gas-discharge plasma properties are substantially determined by electron gas, thus much attention is paid in literary sources to finding its distribution function. In order to calculate EDF in practice, local approximation is usually used when in Boltzmann kinetic equation for isotropic part of EDF the spatial diffusion is neglected in comparison with the electrons energy change in the field and due to collisions with gas atoms and molecules^1–6^. In this case, EDF *f*(*w*, *r*, *t*)

1 $$f(w,r,t)={n}_{e}(r,t){f}_{0}(w,E/p)$$

is factorized as the electron density *n*_*e*_(*r*, *t*), depending on spatial coordinates and time, and EDF *f*_0_(*w*, *E*/*p*), depending on electron kinetic energy *w*, values of local field (*E*/*p*) and other parameters (gas temperature, density of excited particles, etc.) in the given point of space *r*.

Criteria of local approximation applicability in literature are obtained in a standard way based on comparison in kinetic equation (see below Eq. (5)) of terms with derivatives by coordinate with terms including derivatives by energy. It is determined by relation between characteristic plasma size *L* and the electron energy relaxation length *λ*_*ε*_

2 $${\lambda }_{\varepsilon }=\sqrt{2{D}_{r}{\tau }_{\varepsilon }},$$

where *D*_*r*_ = *υλ*/3 is the electron diffusion coefficient, and corresponding time of energy relaxation

3 $${\tau }_{\varepsilon }^{-1}=\delta \nu +\nu \,\ast $$

is determined by energy losses in elastic and inelastic collisions (corresponding frequencies *ν* and ν^*^), *δ* ≪ 1 is the energy exchange factor during quasi-elastic collisions at small electron energy losses per collision ($\delta =\sqrt{2m/M}$ for elastic collisions).

Let us recall that at *λ*_*ε*_ > *L* spatial diffusion occurs much faster than diffusion with respect to energy in electric field. In this case to use of local approximation for EDF is not physically justified and, as has been repeatedly demonstrated in literature, leads to errors (for example, see refs^1–7^). Such EDF is nonlocal, as it is determined by physical property values (primarily by fields) not at the given point, but in the region determined by energy relaxation length (2) *λ*_*ε*_ ≫ *λ* - electron mean free path. Therefore, in order to find such EDF, it is necessary to solve a kinetic equation in variables of both energy and coordinates.

In the limiting case of “full” nonlocality *λ*_*ε*_ ≫ *L*, the energy received by an electron is rapidly redistributed over entire region of space available to it. Since the whole discharge volume is available to the electrons, the ambipolar field of space charge exceeds the heating (external) field and the integral of motion of electrons is total energy (kinetic plus potential energy *ε* = *w* + *eφ*(*r*)), *φ*(*r*) is the potential energy associated with the space charge field^1^. For this reason, the arguments of EDF are *ε*, *r* and kinetic equation takes easier form of two-dimensional diffusion in space of coordinates and energy (see refs^1–6^ for details). For trapped electrons with energy $e < e{\phi }_{w}$-wall potential (potential differences between point (axis) of plasma density maximum and borders) after averaging over discharge volume kinetic equation depends only on *ε*, i.e. comes down to a form formally aligning with local one^1^. At the same time, for electrons with full energy *ε* > *eφ*_*w*_ it is necessary to solve nonlocal kinetic equation in variables as *ε* and *r*.

In its turn, the fulfillment of condition *L* > *λ*_*ε*_ gives occasion to the wide use of local approximation (1) for EDF in practical calculations^1–6^. Local approximation attraction and widespread occurrence are mainly related to computational procedure significant simplification when solving the Boltzmann kinetic equation for *f*_0_, which in this case depends only on one variable (velocity or *w* = *mv*^2^/2 kinetic energy) and has the form of^2–6^

4 $$-\frac{1}{\sqrt{w}}\frac{\partial }{\partial w}(\sqrt{w}{(eE)}^{2}{D}_{r}\frac{\partial {f}_{0}}{\partial w})=St({f}_{0})+St\,\ast \,({f}_{0})$$

In Eq. (4), *E* is an electric field at the given space point, *St*(*f*_0_) and St^*^(*f*_0_) are the integrals of elastic and inelastic collisions, respectively.

It should be noted that the most common and popular fluid models of plasma and gas-discharges are based on the local approximation for EDF (1, 4) calculation. These models, due to their physical visualization and comparative usability, are widely used in practice (see, for example, refs^3,4,8–10^). Based on them currently, the prepared commercial software available for a wide range of researchers allowing to simulate various gas discharge devices, became widely known (see, for example, refs^11,12^). Due to their common usage, these models are constantly improved by considering additional processes and effects. As a result, so far various modifications of fluid description are developed in detail in practice and wide experience of their use is accumulated both at one-dimensional (1D) and two-dimensional (2D) simulation of various gas discharges^13–15^.

Unfortunately, the “reverse side” of fluid models wide use is the fact that the scope is often not discussed at all or interpreted very widely (see, for example, refs^8–15^). Therefore, in practice, these models are often used for conditions where their applicability is doubtful, so it is difficult to assess the reliability of the results obtained.

Analysis shows that the most debating point is an electric field, which is included explicitly both in initial (see below Eq. (5)) and local kinetic equation (4). As an electron responds to total field which exists at the given space point, the full electric field at the given location is included in initial kinetic equation, and, consequently, also in Eq. (4). As a rule, for numeric simulations by various calculating codes^15–17^, that *E* total field, which is found from solution of Poisson’s equation, is substituted into kinetic equation.

By contrast to this, when analyzing the Boltzmann kinetic equation in refs^1,2^ (see also ref.^4^), it was noted that as ambipolar field is also determined by spatial gradients (*E*_*a*_ ≈ *T*_*e*_∇*n*_*e*_/*n*_*e*_). Then when using local approximation for EDF, ambipolar electric field *E*_*a*_ also should be ignored in the local kinetic equation (4). In other words, ambipolar field should be somehow removed (subtracted) from total field, i.e. instead of *E*, you should substitute *E* − *E*_*a*_ difference in local kinetic equation (4).

In its turn, outputs^16–19^ clearly indicate the influence of ambipolar field on EDF formation at plasma volume periphery, where it becomes dominant even when *L* > *λ*_*ε*_ criterion is satisfied (i.e. literary sources^1–6^ predicate the local approximation applicability). Positive discharge column simulations performed both in atomic (argon)^17–23^] and molecular (nitrogen and oxygen)^24^ gases show that at plasma periphery, where *E*_*a*_ ambipolar field (soon or late) exceeds *E*_*h*_ heating field, excitation response increase is observed, due to additional heating of fast electrons exactly by ambipolar field.

Thus, the question of which field should be substituted into local kinetic equation (total *E*, determined from Poisson’s equation, or other than ambipolar *E* − *E*_*a*_) is a debating, concerning which there is still no unequivocal opinion in literature.

The research in this paper has continued^17,18^. It is shown that the local approximation (4) for EDF and other characteristics of electron gas at plasma periphery determination, where ambipolar field is dominant, is not applicable even when *L* > *λ*_*ε*_ criterion is satisfied. In this case, the attempts to substitute both as total *E* and *E* − *E*_*a*_ difference (except ambipolar field) into local kinetic equation are prospectless. Therefore, consistant results at plasma periphery can be obtained only when solving the kinetic equation considering both energy and spatial variables.

## Methods

As is shown in previous papers^17–22,25^, the subject of research was DC discharge positive column (PC). As is known, the heating field in PC is directed in longitudinal (axial) direction, while ambipolar field - in radial direction, i.e. they are perpendicular to each other. This allows dividing their contributions more clearly in EDF spatial profiles formation and other electron characteristics.

Argon was used in a wide range of pressures as power gas, when, according to known criteria (see (2, 3)), both non-local (*λ*_*ε*_ > *L*) and local (*λ*_*ε*_ < *L*) modes of EDF formation are implemented.

A set of plasmachemical reactions in argon includes direct ionization, excitation from the ground state, radiative excitation, step ionization from the metastable state, and Penning ionization (see Table 1). Plasma contains argon atoms in *Ar* ground state, metastable atoms Ar^*^, radiating atoms Ar^**^ and positive ions *Ar*^+^.

**Table 1:** Argon chemistry.

Reaction	Comment
*e* + *Ar* → *e* + *Ar*	momentum transfer^26^
*e* + *Ar* → *e* + *Ar*_m_^*^	metastable state excitation^26^
*e* + *Ar* → *e* + *Ar*_r_^*^	resonant state excitation^26^
*e* + *Ar* → 2*e* + *Ar* +	direct ionization^26^
*e* + Ar^*^ → 2*e* + *Ar* +	stepwise ionization^27^
2Ar^*^ → *Ar* + *Ar*^+^ + *e*	penning ionization^28^

In order to conduct numerical experiments, a computation model which includes a number of related modules in COMSOL Multiphysics environment was developed:

(A) A new kinetic module^25^ was developed in order to calculate 2-dimensional (*r*, *w*) cylindrical symmetrical EDF in discharge positive column. This module includes the Boltzmann equation in coordinate and kinetic energy variables recorded for a one-dimensional positive column (in which the axial heating field is directed perpendicular to radius) in the following form:

5 $$\begin{array}{c}-di{v}_{r}(\upsilon {D}_{r}gra{d}_{r}{f}_{0})+di{v}_{r}(\upsilon e{E}_{a}{D}_{r}\frac{\partial {f}_{0}}{\partial w})+\frac{\partial }{\partial w}(e{E}_{a}\upsilon {D}_{r}gra{d}_{r}{f}_{0})\\ \quad -\frac{\partial }{\partial w}(\upsilon {D}_{r}({E}_{a}^{2}+{E}_{h}^{2})\frac{\partial {f}_{0}}{\partial w})-\frac{\partial }{\partial w}(\upsilon \delta \nu w{f}_{0})=\upsilon S{t}_{0}^{\ast }\end{array}$$

where *div*_*r*_ is a radial divergence component.

(B) A diffusion-drift module, which includes equations for: finding the densities of all neutral and charged particles; drift-diffusion module and Poisson’s equation for field and potential profiles calculation was used.

Balance equation for *Ar*^+^ ions in the form of:

6 $$\frac{d{n}_{i}}{dt}+di{v}_{r}(-{D}_{i}\frac{{\rm{\partial }}{n}_{i}}{{\rm{\partial }}r}+{b}_{i}{E}_{amb}{n}_{i})={R}_{i\text{\_}d}+{R}_{i\text{\_}s}+{K}_{p}{(A{r}^{\ast })}^{2},$$

where *n*_*i*_ - ions *Ar*^+^ density, *D*_*i*_ = (*b*_*i*_*T*_*i*_)/(*e*) - diffusion coefficient for ions, *b*_*i*_ - ions mobility coefficient, *T*_*i*_ - ion temperature (room temperature is taken), *R*_*i*_*d*_ and *R*_*i*_*s*_ - direct and step ionization rate, *K*_*p*_ - Penning ionization constant.

Balance equation for metastable Ar^*^ argon atoms is of the following form:

7 $$\frac{\partial {n}_{m}}{\partial t}+di{v}_{r}(-{D}_{Ar1}\frac{\partial {n}_{m}}{\partial r})={R}_{Ar1\text{\_}{\rm{exc}}}-{Z}_{Ar1\text{\_}deexc}-{R}_{i\text{\_}s}+{K}_{p}{({n}_{m})}^{2}-{K}_{Q}{n}_{e}A{r}_{1},$$

where *n*_*m*_ - density of metastable Ar^*^ argon atoms, *D*_*m*_ - diffusion coefficient for Ar^*^, *R*_Ar1_exc_ - excitation reaction rate, *Z*_Ar1_deexc_ - de-excitation reaction rate, *K*_*Q*_ - extinction reaction constant.

Balance equation for radiating Ar^**^ argon atoms is of the following form:

8 $$\frac{\partial {n}_{r}}{\partial t}+di{v}_{r}(-{D}_{Ar1}\frac{\partial {n}_{r}}{\partial r})={K}_{Q}{n}_{e}A{r}_{1}+{R}_{{\rm{Ar}}2\text{\_}{\rm{exc}}}-{n}_{r}{K}_{rad},$$

where *n*_*r*_ - radiating argon atoms density, *R*_Ar2_exc_ - radiative excitation rate, *K*_*rad*_ - radiative time constant (1*10^6^1/*s*);

Poisson’s equation for *E*_*r*_ =− ∂*φ*/∂*r* radial electric field (where *φ* is a radial potential) has the following form:

9 $$\frac{1}{r}\frac{\partial }{\partial r}(r\,{E}_{r})=\rho /{\varepsilon }_{0},$$

where *ε*_0_ = 8.854 × 10^−12^ F/m is an electric constant and *ρ* = *e*(*n*^+^ − *n*_*e*_) is volume charge density.

(C) Longitudinal (heating) field in *E*_*h*_ PC can be found by means of iterative procedure as a field providing the given *I*_0_ current using the electron density and mobility, both derived from kinetic module.

## Results and Discussion

By using the above self-consistent kinetic model of gas discharge positive column (with Boltzmann equation (5) solution for electrons in energy and radius variables), simulations of gas discharge positive column in argon in a tube of *R* = 1 *cm* radius at a wide pressure range (from 0.3 to 50 Torr) at discharging currents of 1 − 10 *mA* were given. Under investigated conditions, plasma ionization degree is low (<10^−5^) and contribution of electron-electron collisions to EDF formation could be ignored.

**Figure 1:**
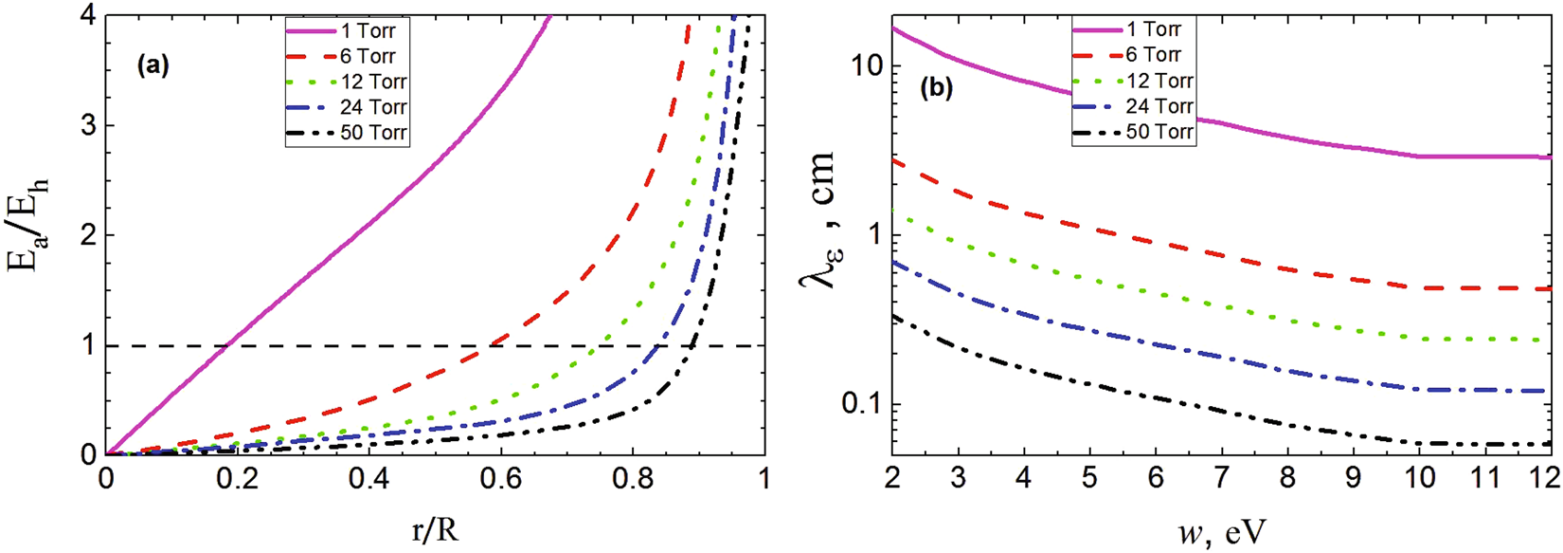
(**a**) Longitudinal (heating) and ambipolar fields in argon at different pressure ratings. (**b**) Values of energy relaxation lengths in elastic energy region.

Simulation results show that at low pressures (less than 1 Torr), when electron energetic relaxation length (2) exceeds the tube radius (*λ*_*ε*_ > *R*), ambipolar field dominates the longitudinal one across entire discharge positive column cross section (see Fig. 1a below). This mode of EDF formation “full” nonlocality was considered in detail in papers^1,2,4^ and to date is well studied both theoretically and experimentally^6^. Since simulations results show the conformity^2,5,6^, this case is notconsidered in this study.

At the same time, at medium and high pressures, when electron energy relaxation length (2) is small (*R* > *λ*_*ε*_) and according to criterion (2) and estimates from^2,4^, a local mode for EDF must be presented and the results of simulations indicate its inapplicability.

**Figure 2:**
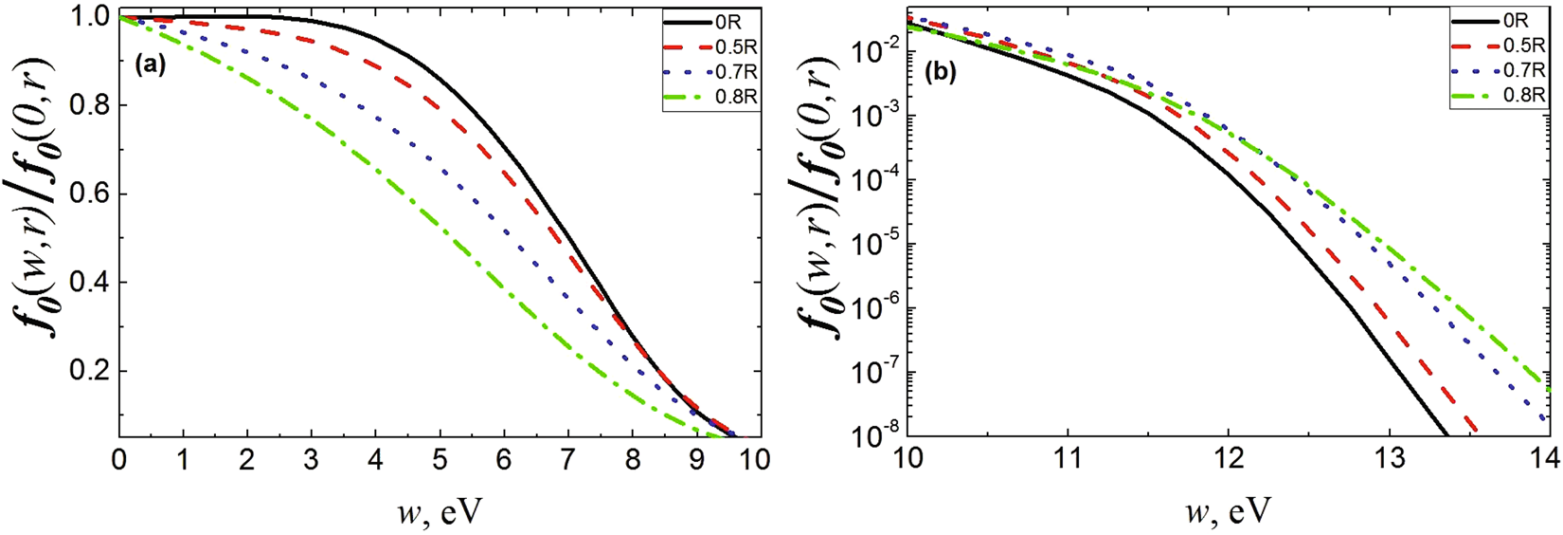
Comparison of EDF at different values of radius for argon at the pressure of 12 Torr (**a**) in elastic region and (**b**) inelastic region. The current is 3 mA.

As an example, Fig. 2 shows the normalized EDFs at various radii for medium pressures of 12 Torr.

As axial heating field *E*_*h*_ in positive discharge column passes constantly across the cross section, then in local case it would be expected the coincidence of normalized EDFs at different radii. Nevertheless, it can be seen that EDF at different radius points do not coincide both in elastic (up to excitation threshold, (*w* < 11.5 *eV*)) and non-elastic (*w* > 11.5 *eV*) regions, i.e. they depend not only on energy, but also on the spatial variable. This means that in these cases the local approximation (4) for electron distribution function calculation at different radius points is not applicable, i.e. EDF is non-local (in the sense that during its calculation it is necessary to use complete kinetic equation (5) in variables of both energy and coordinates).

As preliminary studies have shown (see also refs^17,18^), the main reason for EDF radial dependence occurrence when fulfilling *λ*_*ε*_ > *R* locality condition is the influence of *E*_*a*_ ambipolar field. This field, as is known^2–4^, increases from the plasma center to periphery, and where it (soon or late) begins to exceed the heating field.

In order to illustrate this, according to the results of this simulation Fig. 1a shows the values of ambipolar and heating fields ratios at different pressures. For convenience to estimate by (2), Fig. 1b shows the values of energy relaxation lengths in elastic energy region (${\lambda }_{\varepsilon }=\lambda /\sqrt{\delta }$) which are applied alongside.

It is seen that at low pressures (less than 1 Torr), when *λ*_*ε*_ > *R*, the ambipolar field dominates throughout discharge volume. As noted above, these conditions correspond to “full” nonlocality mode, which was considered in detail earlier in works^2,6^. With increasing pressure (at pressure full length), when *R* > *λ*_*ε*_ opposite inequation is satisfied (when electron energy relaxation length is small) (see Fig. 1b), the boundary of ambipolar field domination over the heating one is shifted to periphery. However, even at high pressures, sooner or later ambipolar field begins to exceed longitudinal field (see also refs^17,18^). According to the results of simulations, roughly from these values of spatial coordinates, radial (spatial) EDF dependences are also observed (see Fig. 2). As can be seen from the local kinetic equation (4), only ambipolar field can give radial dependence (in PC the axial field is homogeneous in radius). This fact argues for substitution in (4) of complete field at the given space point, which was recommended earlier in ref.^24^ for ambipolar field contribution to EDF formation approximate consideration.

It should also be noted that since the losses dominate (play a major role) due to elastic collisions at increased pressures in electrons energy balance, EDF form is sensitive to corresponding collision cross sections behavior related to energy. In particular, in refs^17,23^ it was shown that excitation rate spatial profiles and EDF quick part form are significantly different, depending on whether cross section of electrons elastic scattering increases or decreases: for cross section raising with energy (by the example of argon), the excitation rate was maximum on discharge periphery, while for dropping the cross sections - sharply declined.

**Figure 3:**
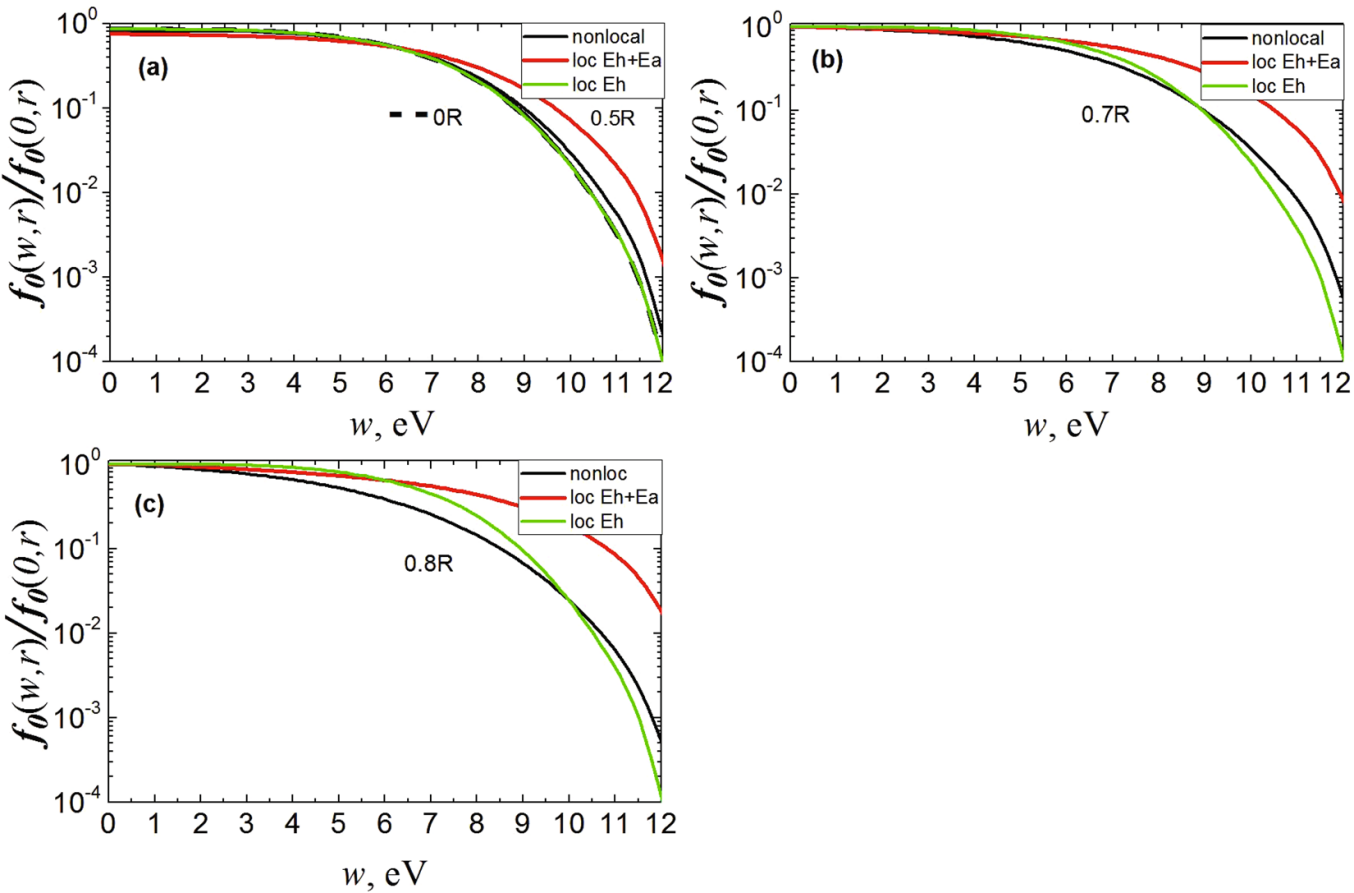
Simulation results comparison (non-local EDF) with local approximation for argon at the pressure of 12 Torr. At the values of radius (**a**) r = 0 R and 0.5 R, (**b**) r = 0.7 R and (**c**) r = 0.8 R. The current is 3 mA.

**Figure 4:**
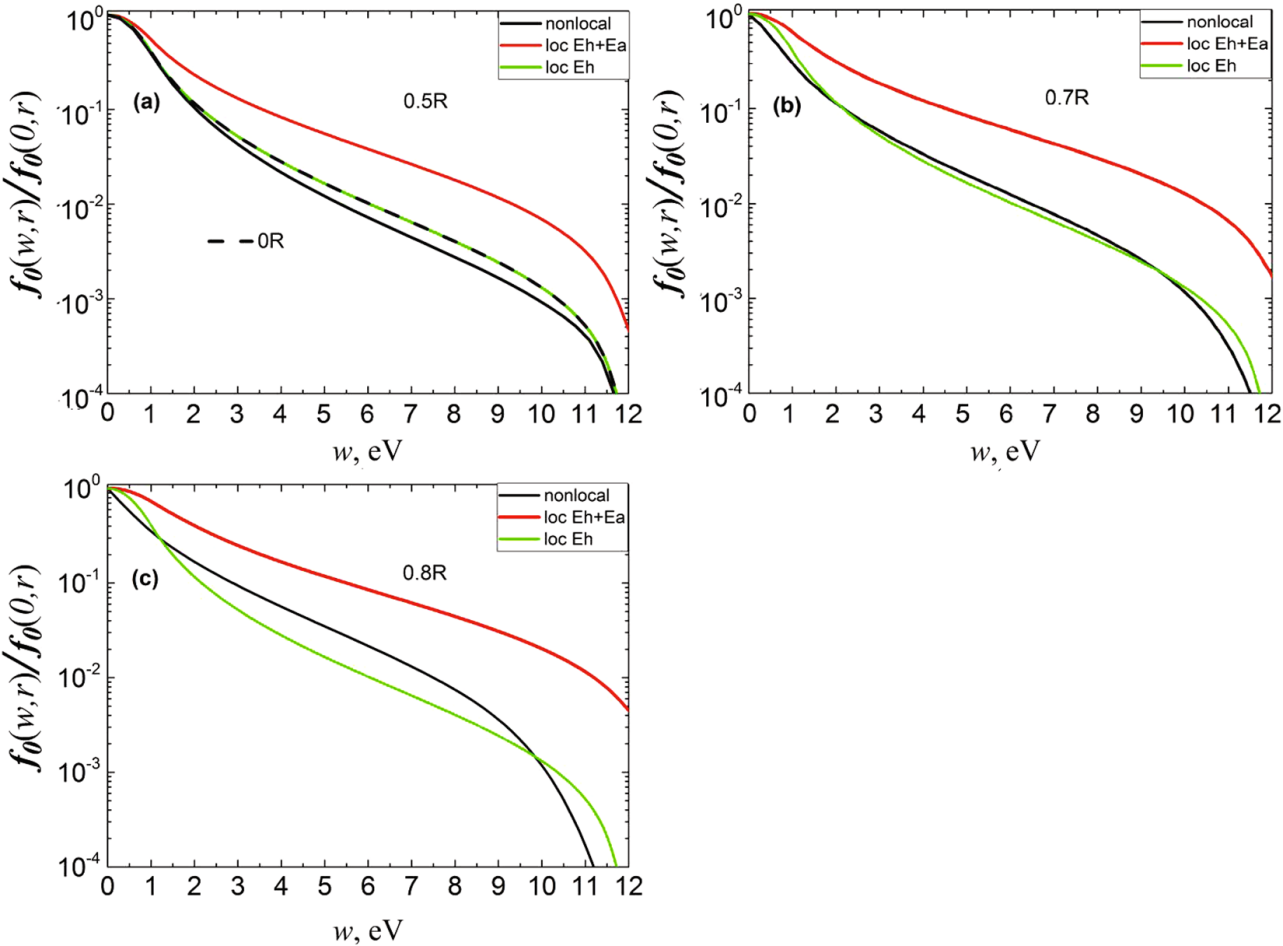
EDF comparison at nonlocal and local approximation for *σ* ~ 1/*w* cross section dependence p = 12 Torr. At the values of radius (**a**) r = 0 R and 0.5 R, (**b**) r = 0.7 R and (**c**) r = 0.8 R. The current is 3 mA.

In order to determine the influence of these factors, the calculations of Boltzmann local equation (4) were performed, with substitution of either longitudinal (*E*_*h*_) or resultant (*E*) field. Figure 3a–c show the results for argon, in which elastic collision cross sections rise from Ramsauer minimum to excitation threshold. Figure 4a–c show the results when *σ* ~ 1/*w* cross sections are dropping.

It can be seen that in near-axial zones where *E*_*a*_ ambipolar field is small in comparison with longitudinal *E*_*h*_ (see Fig. 1a), the results of EDF local calculation correspond closely (coincide) to complete kinetic equation solution. At the same time, regardless of elastic scattering cross sections behavior (rising or dropping with energy) at discharge periphery, where the ambipolar field dominates, a complete kinetic equation solution (5) does not correspond to local calculations in accordance with (4) substitution of both complete field (*E*^2^ = *E*_*a*_^2^ + *E*_*h*_^2^) and one longitudinal *E*_*h*_^2^.

## Conclusion

Thus, at plasma periphery, where ambipolar field exceeds the heating one, even when *R* > *λ*_*ε*_ condition is satisfied, local approximation (4) cannot be used. The reason for this is the fact that non-homogeneous plasma in kinetic equation includes not one, as in local (4), but 3 terms containing electric field (see Eq. (5)). Therefore, depending on conditions (primarily elastic collisions cross sections behavior), various terms of kinetic equation with field can dominate. In such situation the attempts to use local approximation for EDF finding are prospectless and at plasma periphery it is necessary to solve complete (nonlocal) kinetic equation in variables of both energy and coordinates.

Because, the role of the ambipolar field increases near the boundaries of the plasma volume, the strongest effect should be expected in situations where the plasma is supported not by a local field (as discussed above in the positive column of a discharge), but by an external ionizer from the near-electrode layers of the space charge (CCP and ICP discharges, and also a negative-glow plasma with a flat or hollow cathode).
