# Publisher Correction: Ambipolar field role in formation of electron distribution function in gas discharge plasma

**DOI:** 10.1038/s41598-018-20356-7

**Published:** 2018-02-02

**Authors:** Chengxun Yuan, E. A. Bogdanov, A. A. Kudryavtsev, K. M. Rabadanov, Zhongxiang Zhou

**Affiliations:** 1 Department of Physics, Harbin Institute of Technology, Harbin, 150001 China; 2 Saint Petersburg State University, St. Petersburg, 199034 Russia

Correction to: *Scientific Reports* 10.1038/s41598-017-15073-6, published online 03 November 2017

The original HTML version of this Article contained a typographical error in the publication date ‘03 November 2017’ which was incorrectly given as ‘06 November 2017’. This has now been corrected in the HTML version of the Article.

